# Not‐so‐simple nephrectomy: Comparative analysis of radical and simple nephrectomy in a high‐volume tertiary referral center

**DOI:** 10.1111/iju.15330

**Published:** 2023-11-06

**Authors:** Ariadni Papadopoulou, Nicholas Campain, Yasmin Abu‐Ghanem, Nimlan Shanmugathas, Marios Poullas, Faiz Mumtaz, Ravi Barod, Maxine Tran, Axel Bex, Prasad Patki

**Affiliations:** ^1^ Division of Surgery and Interventional Science University College London, Royal Free Hospital London UK; ^2^ The Specialist Centre for Kidney Cancer Royal Free Hospital London UK; ^3^ Department of Urology Royal London Hospital, Barts Health NHS Trust London UK; ^4^ Department of Cell and Developmental Biology University College London London UK; ^5^ Department of Computer Science Neapolis University Pafos Pafos Cyprus

**Keywords:** kidney neoplasms, nephrectomy, nephrostomy, postoperative complications, pyelonephritis

## Abstract

**Objectives:**

Simple nephrectomies can be challenging with significant morbidity. To prove the hypothesis of “not‐so‐simple” nephrectomy, we compared demographics, perioperative outcomes, and complications between simple and radical nephrectomy in a tertiary referral center.

**Methods:**

We analyzed 473 consecutive radical nephrectomies (January 2018–October 2020) and simple nephrectomies (January 2016–October 2020). Univariate and multivariate analysis of perioperative outcomes utilized the Mann–Whitney *U* test, Chi‐squared test, Mantel–Haenszel test of trend, and multiple linear regression. Radical nephrectomies were classified in cT1, cT2a, and cT2b‐T3 subgroups and compared to simple nephrectomies. Minimally invasive and open techniques were compared between the two groups. Infected versus non‐infected simple nephrectomies were compared.

**Results:**

A total of 344 radical and 129 simple nephrectomies were included. Simple nephrectomy was an independent predictor of increased operative time (*p* = 0.001), length of stay (*p* = 0.049), and postoperative complications (*p* < 0.001). Simple nephrectomies had higher operative time (*p* < 0.001), length of stay (*p* = 0.014), and postoperative morbidity (*p* < 0.001) than cT1 radical nephrectomies and significantly more Clavien 1–2 complications than cT2a radical nephrectomies (*p* = 0.001). The trend was similar in minimally invasive operations. However, conversion to open rates was not significantly different. Infected simple nephrectomies had increased operative time (*p* < 0.001), length of stay (*p* = 0.005), blood loss (*p* = 0.016), and intensive care stay (*p* = 0.019).

**Conclusions:**

Patients undergoing simple nephrectomy experienced increased operative time and morbidity. Simple nephrectomy carries higher morbidity than radical nephrectomy in tumors ≤10 cm. Robotic simple nephrectomies may reduce open conversion rates. Postoperative intensive care and enhanced recovery may be essential in simple nephrectomy planning with infected pathology.

**Abbreviations & Acronyms**
BAUS: British Association of Urological Surgeons
HDU: high‐dependency unit
ICU: intensive care unit
MI: minimally invasive
MIS: minimally invasive surgery
PUJO: pelvic‐ureteric junction obstruction
RCC: renal cell carcinoma
RN: radical nephrectomy
SN: simple nephrectomy
TNM: tumor‐node‐metastasis
UTIs: urinary tract infections
XPN: xanthogranulomatous pyelonephritis

## INTRODUCTION

Simple nephrectomy (SN) is performed for the treatment of non‐functioning kidneys, symptomatic and recurrent infection, and associated sequalae such as abscess formation and fistulization.^1^, ^2^

Evidence suggests “simple” nephrectomy is far from simple.^1^, ^3^, ^4^ Inflammatory and fibrotic tissue reaction, such as those associated with xanthogranulomatous pyelonephritis (XPN), can lead to difficulty with surgical tissue planes, making surgery challenging. A contemporary analysis of the British Association of Urological Surgeons (BAUS) national dataset found SN cases to have 1.8 times higher risk of conversion‐to‐open (*p* = 0.0039) and significantly increased rate of transfusions (4.8 vs. 2.8%; *p* = 0.0143) compared to T1 radical nephrectomies (RNs). Intra‐ and postoperative complications were higher in SN cohort (5.2 vs. 3.7% and 11.9 vs. 10%), although the relationship did not reach significance.^4^ Conversely, an online survey of (*n* = 95) urologists reported no difference between urologists' views of outcomes and complications of SN and RN.^5^ Furthermore, Sahai et al. found no significant difference in mental health‐related quality of life of RN and SN patients.^6^ However, there are no reports comparing SN outcomes with outcomes of RN on T2 and T3 renal cell carcinoma (RCC) and no relevant reports including robotic cases or adjusting for confounders with multivariate analysis.

To elucidate whether SN is a negative predictor of perioperative outcomes and complications, we retrospectively compared the intra/peri‐operative outcomes of all SN and RN in a high‐volume institution. This report is the first to adjust for discrepancies in patient demographics and surgical approach between SN and RN with a multivariate investigation. It also includes the first subgroup analysis comparing RN on T2a and T2b‐T3 disease and the only collection of robotically assisted cases in relevant literature, facilitating comparison of SN and RN outcomes within evolving surgical practices.

## METHODS

### Patient population

After obtaining institutional review board approval, we retrospectively analyzed a series of patients undergoing minimally invasive and open nephrectomy in a single institution. Three hundred and forty‐four RNs (January 2018–October 2020) and 150 SNs (January 2016–October 2020) performed in a specialist kidney cancer center, were included in the study. Data on SN were collected over a longer timeframe due to preponderance of RN in a tertiary renal cancer unit to perform a balanced statistical analysis. Partial nephrectomies were excluded.

### Covariates

Baseline data recorded to assess population comparability included age, gender, laterality, BMI, INR, intraoperative creatinine, eGFR, American Society of Anesthesiologists (ASA) score, and indication for surgery. Data were retrospectively retrieved through the prospective patient database, operative notes, inpatient records, discharge summaries, and clinic correspondence. Radiographic tumor size was retrieved from radiology reports.

For subgroup analysis, surgical techniques were grouped into minimally invasive (MI‐robotic and laparoscopic) and open surgery and outcomes for SN and RN were compared by technique.

RN were classified according to tumor size into three groups: Group 1 cT1, Group 2 cT2a, and Group 3 cT2b‐T3 as per the TNM classification^7^ and individually compared to SN. SN was classified further according to pathology as Infected, including recurrent UTIs, abscesses, pyelonephritis, and XPN and non‐infected including atrophic or non‐functioning kidneys secondary to PUJO/hydronephrosis, retroperitoneal fibrosis, pelvic and retro‐vaginal endometriosis, calculus without documented infection, polycystic kidney disease, and iatrogenic trauma and compared.

### Outcome measurements

Outcome variables included operative time, length of hospital stay, estimated blood loss, conversion to open surgery, planned and planned intensive care unit (ICU)/high dependency unit (HDU) stay, readmission rates within 28 days postoperatively, intraoperative, and postoperative complications. Operative time was defined as the total interval between skin incision and skin closure. Postoperative complication up to 28 days postoperatively and their management were recorded and classified according to the Clavien–Dindo classification as minor (Clavien grade 1–2) or major (Clavien grade 3–5) complications.^8^, ^9^

### Statistical analysis

Descriptive statistics of demographics and clinical characteristics including median, and interquartile range were calculated accounting for the non‐normal data distribution.

Univariate analysis utilized the Mann–Whitney U test for quantitative variables, Chi‐squared/Fisher's exact test for nominal categorical variables, and Mantel–Haenszel test of trend for overall postoperative complications. Notice that the Mann–Whitney *U* test used due to non‐normal data distribution does not compare medians and instead considers all datapoints. It tests for the null hypothesis that when two values *X* and *Y*, one from each population, are selected and compared there is equal probability that *X* is greater than *Y* as that *Y* is greater than *X*. Hence, statistically significant difference can be observed with similar medians. In those cases, means are reported for completion.

Multiple linear regression evaluated the independent effect of operation type (SN vs. RN) on perioperative outcomes across all cases after adjusting for age, gender, BMI, preoperative eGFR, laterality, ASA score, and surgical approach. Adjusted beta coefficients, standard error, test statistic, and *p*‐values were recorded. For all tests, a two‐sided *p*‐value <0.05 was considered statistically significant. Nearest neighbor analysis was performed and missing data were completed based on the 23 closest datapoints available. The analysis was carried out with SPSS^10^ and Matlab.^11^

These data were presented at the BAUS 2021 “Global Urology” Virtual Meeting (P12‐9) and the European Association of Urology 2021 Virtual Conference (abstract number: AM21‐4735).

## RESULTS

A total of 344 RNs and 129 SNs were included. Patient demographics are summarized in Table 1.

**TABLE 1:** Patient and clinical characteristics in RN and SN groups (*N* = 473).

	RN (*n* = 344)	SN (*n* = 129)	*p*‐value
Median age at surgery, years (range)	63 (29–90)	48.5 (19–79)	**<0.001** ***
Gender, % (*n*)			
Male	64% (219)	42% (54)	**<0.001** ***
Female	36% (125)	58% (76)	
Median ASA score (range)	2 (1–4)	2 (1–3)	**0.038** *
Laterality, % (*n*)			
Left	53% (181)	56% (62)	0.529
Right	47% (161)	44% (48)	
Median BMI (range)	28 (15–47)	26 (15–58)	**0.013** *
Median preoperative sCr, mg/dL (range)	82 (36–1086)	88.5 (53–856)	**0.040** *
Median preoperative eGFR (range)	80 (15–90)	72 (15–90)	**0.032** *
Initial MI approach, % (*n*)	91% (313)	95% (125)	0.127
Transperitoneal approach, % (*n*)	97% (333)	87% (113)	**<0.001** ***
Robotic approach (vs. laparoscopic), % (*n*)	60% (207)	53% (69)	

*Note*: *p*‐value < 0.05 was considered significant. *p*‐values presented in bold are statistically significant.

* *p* < 0.05.

*** *p* < 0.001.

SN was associated with significantly younger age at time of surgery (63 vs. 48.5; *p* < 0.001), female gender (58% vs. 36%; *p* < 0.001) and lower ASA score (*p* = 0.038) and eGFR (*p* = 0.032). MIS approach was used for 91% of RN and 95% of SN (*p* = 0.127). Robotic cases accounted for 60% of RN and 53% of SN.

### Univariate and multivariate analysis of perioperative outcomes in RN versus SN

#### Operative time

**FIGURE 1:**
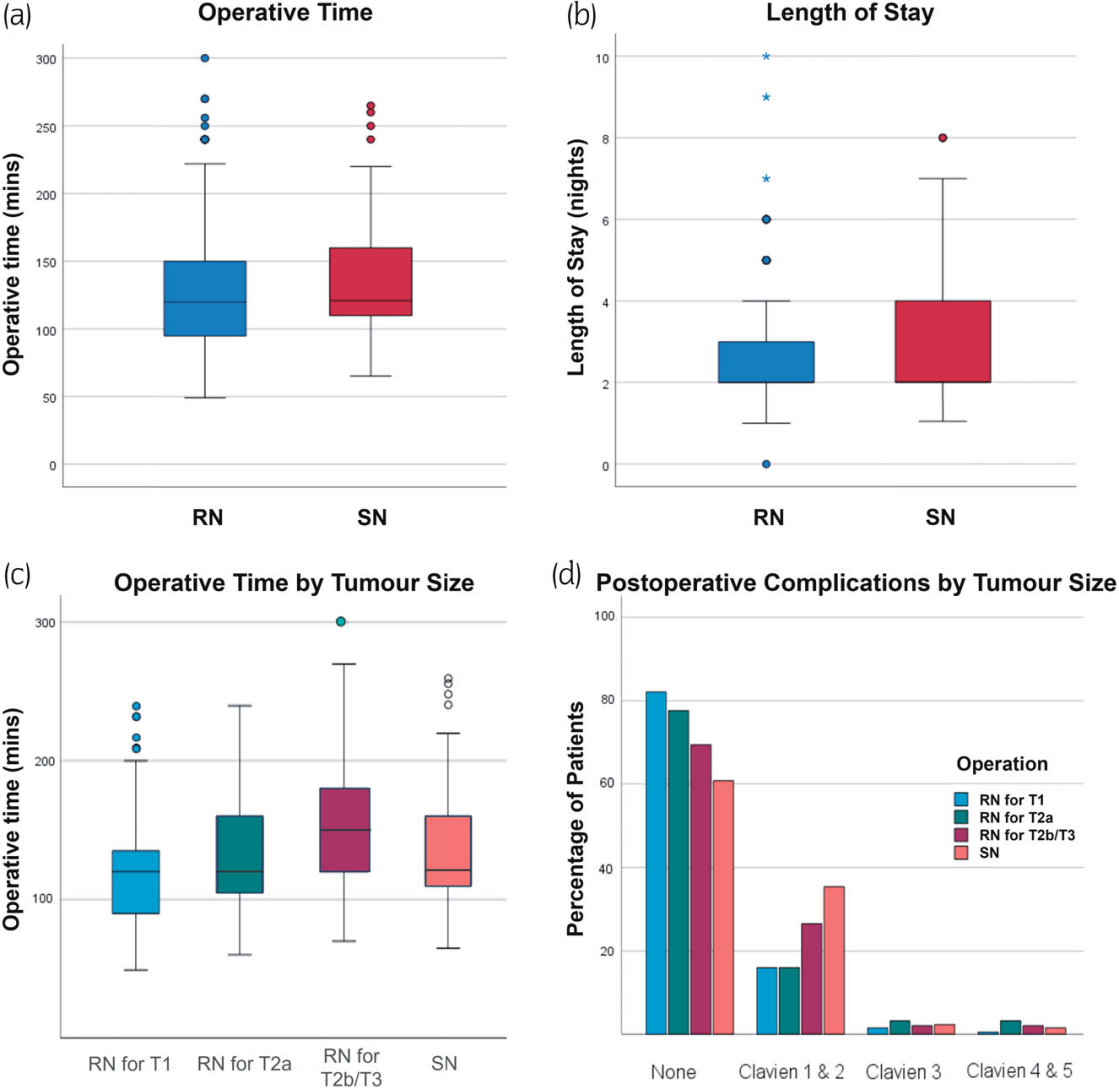
Data distribution for select intra‐ and postoperative outcomes of radical (RN) and simple nephrectomies (SN). (a) Box plot of operative time across all cases. (b) Box plot of length of stay across all cases. The median length of stay is equal to the 25th percentile for both RN and SN (2 nights). (c) Box plot of operative time across all cases classified by RN tumor size. (d) Bar graph of postoperative complication frequency in SN and RN according to tumor size.

In the univariate analysis, SN was associated with significantly higher operative time compared to RN (mean: 136.42 min vs. 126.91 min, *p* = 0.023; Table 2; Figure 1a). The difference remained significant in multivariate analysis (*p* = 0.001; Table 3). Factors associated with longer operative time on multivariate analysis included higher BMI (*p* < 0.001), ASA score 3 (vs. ASA 1) (*p* = 0.036), and open surgical approach (*p* < 0.001; Table 3).

**TABLE 2:** Perioperative outcomes for all cases in the RN and SN groups (*N* = 473).

	RN (*n* = 344)	SN (*n* = 129)	*p*‐value
Median operative time, minutes (range)	120 (49–300)	121 (65–265)	**0.023** *
Median length of stay, nights (range)	2 (0–30)	2 (1–41)	0.106
Median blood loss, mL (range)	20 (0–2500)	40 (0–1300)	0.444
Conversions, % (*n*)	2% (8)	2% (3)	1.000
HDU/ICU admissions, % (*n*)	7% (23)	6% (7)	0.729
Unplanned HDU/ICU Admissions, % (*n*)	57% (13)	71% (5)	0.481
Readmissions, % (*n*)	2% (8)	6% (6)	0.064
Intraoperative complications, % (*n*)	3% (9)	4% (5)	0.371
Postoperative complications, % (*n*)			
Minor complications (Clavien grade 1–2)	17% (60)	35% (46)	**<0.001** ***
Major complications (Clavien grade 3–5)	3% (12)	4% (5)	0.788
Overall	21% (72)	39% (51)	**0.006** **
Transperitoneal approach, % (*n*)	97% (333)	87% (113)	**<0.001** ***

*Note*: Significance may be observed where median values are similar due to differences in data distribution between the groups. The Mann–Whitney *U* test compares rank sums. *p*‐value <0.05 was considered significant. *p*‐values presented in bold are statistically significant.

* *p* < 0.05.

** *p* < 0.01.

*** *p* < 0.001.

**TABLE 3:** Multiple linear regression of select outcomes after SN and RN (*n* = 473).

	Adjusted coefficient	Adjusted LL 95% CI	Adjusted UL 95% CI	*p*‐value
Operative time				
Age	0.205	0.065	0.346	0.144
BMI	1.204	0.861	1.547	**<0.001** ***
eGFR	0.0510	−0.051	0.153	0.616
Female gender	−7.972	−12.358	−3.587	0.070
Left laterality	−6.183	−10.523	−1.843	0.155
MI (vs. open)	−24.168	−30.487	−17.849	**<0.001** ***
ASA score 2 (vs. 1)	4.6754	−0.037	9.387	0.322
ASA score 3 (vs. 1)	14.735	7.730	21.739	**0.036** *
ASA score 4 (vs. 1)	47.494	7.828	87.160	0.232
Radical nephrectomy (vs. simple nephrectomy)	−15.352	−20.051	−10.653	**0.001** **
Length of stay				
Age	0.012	0.001	0.023	0.277
BMI	0.005	−0.022	0.032	0.844
eGFR	−0.02	−0.028	−0.012	**0.011** *
Female gender	0.086	−0.257	0.429	0.802
Left laterality	−0.008	−0.348	0.332	0.981
MI (vs. open)	−3.224	−3.719	−2.73	**<0.001** ***
ASA score 2 (vs. 1)	0.413	0.045	0.782	0.263
ASA score 3 (vs. 1)	1.7	1.152	2.248	**0.002** **
ASA score 4 (vs. 1)	0.571	−2.533	3.675	0.854
Radical nephrectomy (vs. simple nephrectomy)	−0.725	−1.093	−0.357	**0.049** *
Estimated blood loss				
Age	1.268	0.401	2.135	0.144
BMI	4.712	2.596	6.829	**0.026** *
eGFR	0.191	−0.436	0.818	0.761
Female gender	−7.158	−34.238	19.922	0.792
Left laterality	−20.367	−47.164	6.43	0.448
MI (vs. open)	−442.964	−481.983	−403.945	**<0.001** ***
ASA score 2 (vs. 1)	−17.314	−46.41	11.781	0.552
ASA score 3 (vs. 1)	48.403	5.153	91.653	0.264
ASA score 4 (vs. 1)	162.693	−82.241	407.628	0.507
Radical nephrectomy (vs. simple nephrectomy)	20.437	−8.581	49.454	0.482
Minor complications				
Age	−0.002	−0.004	−0.001	0.128
BMI	0.0001	−0.003	0.004	0.973
eGFR	0.001	−0.0005	0.002	0.592
Female gender	0.023	−0.022	0.069	0.608
Left laterality	−0.045	−0.091	−0.0001	0.317
MI (vs. open)	−0.109	−0.175	−0.043	0.098
ASA score 2 (vs. 1)	0.088	0.039	0.137	0.073
ASA score 3 (vs. 1)	0.159	0.086	0.232	**0.03** *
ASA score 4 (vs. 1)	−0.075	−0.488	0.339	0.857
Radical nephrectomy (vs. simple nephrectomy)	−0.158	−0.207	−0.109	**0.001** **
Major complications				
Age	0.0002	−0.0004	0.001	0.705
BMI	0.002	0.00002	0.003	0.305
eGFR	−0.001	−0.001	−0.0005	**0.049** *
Female gender	−0.028	−0.048	−0.007	0.175
Left laterality	0.027	0.007	0.047	0.178
MI (vs. open)	−0.068	−0.097	−0.039	**0.021** *
ASA score 2 (vs. 1)	−0.007	−0.029	0.015	0.752
ASA score 3 (vs. 1)	0.002	−0.03	0.035	0.94
ASA score 4 (vs. 1)	−0.049	−0.233	0.134	0.788
Radical nephrectomy (vs. simple nephrectomy)	−0.019	−0.041	0.002	0.374
Overall postoperative complications				
Age	−0.002	−0.004	−0.0005	0.195
BMI	0.002	−0.002	0.005	0.641
eGFR	−0.0004	−0.001	0.001	0.747
Female gender	−0.004	−0.052	0.044	0.932
Left laterality	−0.018	−0.066	0.029	0.699
MI (vs. open)	−0.177	−0.246	−0.108	**0.011** *
ASA score 2 (vs. 1)	0.081	0.03	0.133	0.114
ASA score 3 (vs. 1)	0.161	0.085	0.238	**0.035** *
ASA score 4 (vs. 1)	−0.124	−0.557	0.309	0.775
Radical nephrectomy (vs. Simple nephrectomy)	−0.177	−0.228	−0.126	**<0.001** ***

*Note*: *p*‐value < 0.05 was considered significant. *p*‐values presented in bold are statistically significant.

* *p* < 0.05.

** *p* < 0.01.

*** *p* < 0.001.

#### Estimated blood loss, intraoperative complications, and conversions to open procedure

Blood loss did not differ significantly between the groups (*p* = 0.482) and the only independent predictors of increased blood loss were higher BMI (*p* = 0.026) and open surgical approach (*p* < 0.001; Table 3). There were 9 (3%) intraoperative complications in the RN and 5 (4%) in the SN group (*p* = 0.371). Conversions to open were noted in eight RN (2%) and three SN (2%) with no significant difference between the groups (*p* = 1.000; Table 2).

#### HDU/ICU admission rates and readmission rates

There were 23 (7%) HDU/ICU admissions in the RN and seven (6%) in the SN group with no significant difference (*p* = 0.729; Table 2). Ten (43%) RN HDU/ICU admissions were planned compared to only two SN HDU/ICU admissions (29%) (*p* = 0.481). Independent predictors associated with increased risk of HDU/ICU admission were open surgical approach (*p* < 0.001) and ASA score 3 (*p* = 0.007; Table 3). Despite readmission rates being higher in the SN cohort (6% vs. 2%) due to higher rates of wound infection, acute kidney injury, and postoperative ileus, the difference was not statistically significant (*p* = 0.064; Table 2).

#### Postoperative complications

The postoperative complication rate following SN was significantly higher than in the RN group in univariate analysis (39% vs. 21%; *p* = 0.006, Table 2). SN remained an independent predictor of increased postoperative complications on multivariate analysis (*p* < 0.001, Table 3). ASA score 3 (*p* = 0.002) and open surgical approach (*p* < 0.001) were also independent predictors of increased postoperative complications (Table 3).

Minor postoperative complication rate was significantly higher in the SN group in univariate (35% vs. 17%; *p* < 0.001, Table 2) and multivariate analysis (*p* = 0.001, Table 3) with ASA score 3 being the other independent predictor of minor complications (*p* = 0.030). Postoperative neuropraxia‐related complications such as numbness and referred pain were significantly more common in the SN cohort (10 vs. 2 events; *p* < 0.001).

Major postoperative complication rates remained low in both groups (3% vs. 4%, *p* = 0.374) and were associated with lower eGFR (*p* = 0.049) and open surgical approach (*p* = 0.020) on multivariate assessment (Table 3). Only one perioperative mortality was noted in the SN group. The patient suffered from ischemic colitis, subsequently succumbing to complications. Detailed postoperative complications are displayed in Table S1.

#### Length of stay

The mean length of stay was longer in the SN cohort (mean: 3.43 vs. 2.92 days) (Table 2; Figure 1b). While this difference was not significant in univariate analysis, operation type was recognized as a significant predictor of length of stay in the multivariate model (*p* = 0.049). Low eGFR (*p* = 0.011), ASA score 3 (*p* = 0.002), and open surgical approach (*p* < 0.001) were independent predictors of prolonged length of stay (Table 3).

### Subgroup analysis

#### Tumor size

When comparing the SN and RN Group 1 (cT1) the SN cases were associated with significantly higher operative time (Mean: 136 min vs. 117 min; *p* < 0.001), length of stay (Median = 3.43 vs. 2.69 days; *p* = 0.014) and more overall postoperative complications (39% vs. 17%; *p* < 0.001; Figure 1c). Clavien 1–2 complications were significantly more common in the SN group than in RN Group 1 (35% vs. 16%; *p* < 0.001) but no difference was noted in major complications. When comparing the SN and RN Group 2 (cT2a), SN was associated with significantly higher rates of Clavien 1–2 complications (35% vs. 16%; *p* = 0.001; Figure 1d). RN Group 3 (cT2b‐T3) had significantly higher blood loss than SN (mean: 343 mL vs. 75 mL; *p* < 0.001; Table 4; Table S2).

**TABLE 4:** Perioperative outcomes of SN and RN groups according to tumor size.

	SN (*n* = 129)	RN Group 1 (*n* = 201)	SN vs. RN1 *p*‐value	RN Group 2 (*n* = 94)	SN vs. RN2 *p*‐value	RN Group 3 (*n* = 49)	SN vs. RN3 *p*‐value
Median operative time, minutes (range)	121 (65–265)	120 (49–250)	**<0.001** ***	120 (60–240)	0.717	150 (70–300)	0.066
Median length of stay, nights (range)	2 (0–41)	2 (0–30)	**0.014** *	2 (1–21)	0.294	3 (1–27)	0.136
Median blood loss, mL (range)	40 (0–1300)	20 (0–2000)	0.186	50 (0–2500)	0.221	200 (5–2000)	**<0.001** ***
Minor complications (Clavien grade 1–2), % (*n*)	35% (46)	16% (32)	**<0.001** ***	16% (15)	**0.001** **	27% (13)	0.261
Major complications (Clavien grade 3–5), % (*n*)	4% (5)	1% (2)	0.323	3% (3)	0.794	6% (3)	1.000
Overall	39% (51)	17% (34)	**<0.001** ***	19% (18)	**0.039** *	33% (16)	0.532

*Note*: Significance may be observed where median values are similar due to differences in data distribution between the groups. The Mann–Whitney *U* test compares rank sums. *p*‐value <0.05 was considered significant. *p*‐values presented in bold are statistically significant.

* *p* < 0.05.

** *p* < 0.01.

*** *p* < 0.001.

#### MIS RN versus MIS SN

For MIS approach, SN cases were associated with significantly higher operative time (*p* = 0.010) and length of stay (*p* = 0.028) compared to MI RN (Table 2). Minor complications following MIS SN were significantly higher than in the MIS RN group (31% vs. 15%; *p* < 0.001), however, major complication rates were similar (Table 5; Table S3).

**TABLE 5:** Subgroup analysis of select perioperative outcomes by surgical approach and SN pathology.

	MI RN (*n* = 313)	MI SN (*n* = 123)	*p*‐value
Median operative time, minutes (range)	120 (49–270)	120 (65–265)	**0.010** *
Median length of stay, nights (range)	2 (0–16)	2 (0–41)	**0.028** *
Conversions, % (*n*)	3% (8)	2% (3)	1.000
Minor complications, % (*n*)	15% (48)	31% (39)	**<0.001** ***
Major complications, % (*n*)	2% (6)	3% (4)	0.481
Overall complications, % (*n*)	17% (54)	35% (43)	**<0.001** ***

	Open RN (*n* = 34)	Open SN (*n* = 6)	*p*‐value
Median operative time, minutes (range)	150 (70–300)	135 (90–185)	0.350
Median length of stay, nights (range)	4 (2–30)	4 (3–6)	0.727
Median blood loss, mL (range)	500 (10–2000)	100 (10–400)	**0.015** *
HDU/ICU admissions, % (*n*)	35% (12)	0% (0)	0.153
Minor complications, % (*n*)	26% (9)	50% (3)	0.341
Major complications, % (*n*)	6% (2)	0% (0)	1.000
Overall complications, % (*n*)	32% (11)	50% (3)	0.740

	Infected SN (*n* = 50)	Non‐infected SN (*n* = 79)	*p*‐value
Median operative time, minutes (range)	150 (82–265)	120 (65–260)	**<0.001** ***
Median length of stay, nights (range)	3 (0–41)	2 (1–16)	**0.005** **
Median blood loss, mL (range)	50 (0–1300)	20 (0–650)	**0.016** *
HDU/ICU admissions % (*n*)	12% (6)	1% (1)	**0.019** *
Minor complications, % (*n*)	36% (18)	35% (28)	0.908
Major complications, % (*n*)	6% (3)	3% (2)	0.372
Overall complications, % (*n*)	42% (21)	38% (30)	0.811

*Note*: *p*‐value < 0.05 was considered significant. *p*‐values presented in bold are statistically significant.

* *p* < 0.05.

** *p* < 0.01.

*** *p* < 0.001.

#### Open RN versus open SN

Open RN were associated with significantly higher median blood loss than open SN cases (Table 5; Table S3). In the open SN group, higher proportion of minor complications (50% vs. 26%) were observed while major complications were more frequent in the open RN cohort (6% vs. 0%) but the trends did not reach significance (*p* = 0.341; *p* = 1.000).

#### Infected versus non‐infected SNs

Of the 129 SNs, 50 (38.8%) were classified as infected and 79 (61.2%) non‐infected (Table S4). Infected SN was associated with significantly higher operative time (150 vs. 120; *p* < 0.001), length of stay (3 vs. 2; *p* = 0.005), blood loss (50 vs. 20; *p* = 0.016), and unplanned HDU/ICU admissions (6 vs. 1; *p* = 0.019; Table 5; Table S5). There was no significant difference in intraoperative and overall postoperative complications, open conversions, and readmissions between the groups.

## DISCUSSION

In this large single‐center study, we have compared perioperative outcomes of SNs and RNs. Our cohort contains the largest collection of robotically assisted operations and the first subgroup analysis of RN on T2 and T3 RCC in the literature (Table 6). A summary of negative intra‐ and postoperative outcome predictors with corresponding recommendations to mitigate them is displayed in Table 7.

**TABLE 6:** Summary of literature comparing SN and RN intraoperative and postoperative outcomes.

Study, setting, sample size	Aim	Study design	Patients (*n*)		Operation	Statistical tools	Findings	Limitations
			SN	RN				
Keeley et al., 1998; Scotland; *n* = 100	To identify risk factors for complications and conversion to open surgery	Cohort	79	21	Retroperitoneal laparoscopic nephrectomy or nephroureterectomy^a^	Nil	Minor postoperative complication rates were higher in SN cases (16% vs. 10%) with a higher rate of major complication in RN (10% vs. 3%). Conversions to open surgery were only noted in inflammatory SN (12% vs. 0%). Operative time and length of stay were highest in the RN group, followed by the inflammatory SN and non‐inflammatory SN cohorts.	Dataset from 1992 to 1997, small sample size, and no analysis of statistical significance were performed.
Permpongkosol et al., 2007; USA; *n* = 2775	To assess the complications of all urological laparoscopic surgery types at a single high‐volume center	Case–control	186	549	Laparoscopic radical and simple nephrectomy^a^	Nil	SN had an increased conversion rate (6% vs. 3%) and median length of stay both with (3 vs. 2 days) and without complications (5 vs. 3 days) compared to RN. RN recorded higher postoperative complication rates (14% vs. 10%).	Dataset from 1993 to 2005, and no analysis of statistical significance was performed.
Connolly et al., 2011; Ireland; *n* = 215	To assess the validity of the “simple” nephrectomy terminology by comparing intra‐ and post‐operative complications of SN and RN	Case–control	89	126	Open RN and SN through lateral/flank incision^a^	Fisher's exact test, linear models	SN was associated with shorter mean operative time (*p* = 0.002), lower blood loss (*p* = 0.472), conversions to open (*p* = 0.087) and shorter length of stay (*p* < 0.001).	Dataset from 1998 to 2002, small sample size, no multivariate analysis, no subgroup analysis by tumor size or etiology and only open approach assessed.
Zelhof et al., 2016; United Kingdom; *n* = 2188	To summarize the UK urologists' practice of nephrectomy for benign disease including outcomes and complications	Case–control	1093	1095	Open and laparoscopic SN and RN on T1 disease	Fisher's exact test	SN was associated with increased conversion to open rates (*p* = 0.004) and higher rate of intra‐ and postoperative complications, but the difference was non‐significant (*p* = 0.097, *p* = 0.171).	No multivariate analysis, no subgroup analysis on T2 and T3 disease, no robotic cases included.
Keshavamurthy et al., 2022; India; *n* = 114	To assesses the validity of the “simple” nephrectomy terminology by comparing intra‐ and postoperative SN and RN outcomes	NS	82	32	Open SN for benign renal disease and open RN for T1/2	Fisher exact test and Mann–Whitney U test	Mean operative time (*p* = 0.08), postoperative complications (*p* = 0.50), length of hospital stay (*p* = 0.09), blood loss >500 mL (*p* = 0.75) were increased in SN.	Small sample size, no multivariate analysis, no subgroup analysis by tumor size or etiology, only open approach assessed.

Abbreviations: RN, radical nephrectomy; SN, simple nephrectomy.

^a^ No available information on radical nephrectomy tumor stage.

**TABLE 7:** Independent negative predictors of intraoperative and postoperative outcomes in SN and RN with corresponding recommendations to mitigate negative outcomes based on the authors' experience.

Outcome	Negative predictors	Recommendations
Operative time	Open surgical technique (vs. MI) ASA score 3 (vs. 1) Higher BMI Infected pathology	MIS approach was associated with reduced operative time in SN and RN. Lateral position with nearside trend usually displaces the large abdominal pannus in high BMI, facilitating the MIS approach. We favor robotic SN and recommend moving the ports more medially to allow for the large retroperitoneal fat and using Bariatric (4th arm) port in patients with pronounced hip mitigating conversion to open.
Length of stay	Open surgical technique (vs. MI) ASA score 3 (vs. 1) Decreased eGFR Infected pathology	MIS approach was associated with reduced length of stay. MIS vs. open approach selection can be facilitated by preoperative imaging and in select cases 3D reconstruction of vasculature. Preoperative optimization (antibiotics) and enhanced recovery mitigates against sepsis and high morbidity.
Estimated blood loss	Open surgical technique (vs. MI) Higher BMI Infected pathology	For safe vascular pedicle management, we recommend performing simultaneous anterior, lateral, and posterior dissection with psoas slide and kidney lift maneuver with all modes of vascular control and repair (port length clipped and free Prolene and Vicryl stitch, Vicryl ligature, Scanlon vascular clamps, Hem‐O‐Loc clips, vascular stapler and laparoscopic Satinsky clamp) available in case of emergency to reduce blood loss and conversion rates. 3D reconstruction of vasculature is also recommended for select patients.
HDU/ICU admissions	Infected pathology	Wound pain/infection constituted the most common complication in both cohorts. We recommend the removal of stent preoperatively. However, if not removed care should be taken to not spill infected urine during intra‐operative ureterotomy. Avoid traction and thermal injury to bowel with expert assistant.
Minor complications	ASA score 3 (vs. 1)	Difficult patient and disease criteria should be identified, discussed in planning (ITU/HDU stay), and treated early. This facilitates availability of experienced assistance, regular surgical team, and multi‐speciality back up.
Major complications	Open surgical technique (vs. MI) Decreased eGFR	

Abbreviations: ASA, American Society of Anesthesiologists; BMI, body mass index; eGFR, estimated Glomerular Filtration Rate; HDU, high dependency unit; ICU, intensive care unit; MI, minimally invasive; RN, radical nephrectomy; SN, simple nephrectomy.

Patients in the SN group were younger, more often female, with a lower ASA score. This may reflect higher rates of recurrent UTI and pyelonephritis in younger women and early presentation with non‐functioning kidney due to long‐standing PUJO in younger population. Atrophic kidneys in SN cohort accounts for lower pre‐operative eGFR. Renal cancer is commonly associated with obesity,^12^ hence lower BMI in the SN group is not surprising.^13^, ^14^

SN cases were associated with significantly higher operative time, as previously reported.^15^, ^16^ This may be attributed to inflammatory adhesions, fibrotic and dense tissue, and infection with edematous friable tissue making intraoperative dissection and anatomical structure differentiation challenging, leading to longer operative duration. However, the median operative time was similar in SN for infected pathology and RN in T2b and above disease. MI approach was an independent predictor of decreased operative time as previously reported,^17^ likely due to case selection bias. When comparing MI SN and RN, SN cases were still associated with significantly higher operative time.

Zelhof et al. report a higher conversion rate to open surgery in laparoscopic SN compared to RN.^4^ Permpongkosol et al. have also reported higher conversion rates for SN.^18^ In our patient cohort, there was no significant difference in conversion to open surgery between the MI SN and MI RN groups (2% vs. 3%), with the majority of MI SN performed robotically compared to the BAUS nationwide dataset (53% vs. 0%, respectively). However, our overall postoperative SN complication rate across all Clavien–Dindo grades was 35% and higher than that reported by BAUS and Keeley et al. (11.9% and 18%, respectively).^4^, ^19^ This might be due to centralization of technically difficult cases and high‐morbidity patients declined surgery elsewhere.

Estimated blood loss did not significantly differ between RN and SN. Open surgical approach was an independent predictor of increased blood loss, with increased blood transfusion rates reported in literature.^20^ Higher BMI was also associated with increased blood loss as previously reported^15^ due to increased visceral fat resulting in significantly longer operative time.^15^ As such, area of visceral fat measured by CT scan provides a more accurate blood loss measure than BMI.^21^ RN for cT2b and above had significantly higher blood loss than SN. Tumor size >10 cm and venous involvement was the critical point beyond which RNs become technically more challenging than SNs.

The only independent ICU admission predictors were open approach and ASA score 3, both associated with increased risk of postoperative complications and ICU admissions in RN in the literature.^22^, ^23^ However, only 2 of 7 ITU admissions in SN group were planned indicating an unanticipated need for higher monitoring and support in SN postoperatively. There was a significantly higher operative time, length of stay, blood loss, and HDU/ICU stay observed in our infected SN cohort compared to non‐infected SN group.

SN (vs. RN) was an independent predictor of higher postoperative complications even after adjusting for differences in age, gender, BMI eGFR, ASA score, and surgical approach between the groups. Keshavamurthy et al. also reported a higher postoperative complication rate of 32.9% in SN patients compared to 25% in RN with a preponderance of minor complications, although the results did not reach significance.^16^ ASA score 3 was an independent predictor of increased minor complications on a background of patient comorbidities as discussed in other publications.^24^, ^25^

More patients in the SN cohort experienced postoperative neuropathy‐related complications such as numbness and referred pain than in the RN cohort (*p* < 0.001). Although reversible, this finding should be communicated to patients to better inform counseling during the consent process. A possible explanation is genitofemoral nerve injury.^26^, ^27^, ^28^ Difficult posterior renal plane dissection and trauma to the genitofemoral nerve may be more likely during SN due to adherent or inflammatory tissue around the psoas muscle perimysium, for example, in patients with XPN or emphysematous pyelonephritis with multiple percutaneous drainage tube insertions.

Open surgical approach was an independent predictor of postoperative complications, including major complications. Zelhof et al. reported higher complication rates in open SN,^4^ however, no analysis of significance was performed making open surgery a novel independent predictor of major postoperative complications in our study. The selection of challenging cases for open nephrectomy may justify this. However, frequent utilization of robotic interface and low conversion rates limit the use of open technique leading to reduced opportunities for surgical training.

To our knowledge, this is first report comparing SN outcomes with RN for T1, T2a, and T2b‐T3 RCC. SN was associated with significantly higher operative time, length of stay, and Clavien 1–2 complications compared to RN for T1 tumors as reported by Zelhof et al.^4^ When compared with RN for cT2a, the SN group still had significantly higher Clavien 1–2 complication rate.

Our study is limited by its retrospective design. However thorough recording of complications was achieved by using a prospectively collected database and confirmed by sourcing multiple documents pertaining to the patient pathway. Any complications in patients repatriated to referring trusts were actively recorded and those needing further treatments were transferred to the center.

In conclusion, despite SN being carried out in younger patients, they have longer operative times, LOS and Clavian 1–2 complications compared to RN for cT1 tumors. Although the postoperative complication rate is higher in SN compared to cT2a RN, RN for tumors greater than 10 cm or for cT2b and above stage have higher blood loss. The overall readmission rates and unplanned transfers to HDU remain high in SN compared to all RN. MI surgery (predominantly robotic) remains a safe option for SN in infected or non‐infected pathology with minimal conversion to open. When compared to RN, SN carries similar or greater surgical morbidity to patients, hence postoperative intensive care and enhanced recovery planning may be essential, particularly with infected pathology.

## AUTHOR CONTRIBUTIONS

**Ariadni Papadopoulou:** Investigation; Writing—original draft; Methodology; Validation; Visualization; Writing—review & editing; Software; Data curation; Project administration; Formal analysis. **Nicholas Campain:** Investigation; Writing—original draft; Methodology; Validation; Writing—review & editing. **Yasmin Abu‐Ghanem:** Writing—review & editing; Methodology; Investigation; Validation. **Nimlan Shanmugathas:** Data curation; Writing—review & editing; Methodology; Validation. **Marios Poullas:** Formal analysis; Software; Methodology; Validation; Investigation. **Faiz Mumtaz:** Investigation; Writing—review & editing; Validation; Methodology. **Ravi Barod:** Investigation; Validation; Methodology; Writing—review & editing; Supervision. **Maxine Tran:** Investigation; Validation; Writing—review & editing; Methodology. **Axel Bex:** Investigation; Methodology; Validation; Writing—review & editing. **Prasad Patki:** Conceptualization; Investigation; Writing—original draft; Methodology; Validation; Writing—review & editing; Supervision.

## CONFLICT OF INTEREST STATEMENT

The authors declare no conflict of interest.

## APPROVAL OF THE RESEARCH PROTOCOL BY AN INSTITUTIONAL REVIEWER BOARD

This study was approved by the Local Reviewer Board of Royal Free Hospital, London, United Kingdom.

## INFORMED CONSENT

N/A.

## REGISTRY AND THE REGISTRATION NO. OF THE STUDY/TRIAL

N/A.

## ANIMAL STUDIES

N/A.

## Supporting information


Table S1–S5

## Data Availability

The datasets generated during and/or analyzed during the current study are available from the corresponding author on reasonable request.
