# Supplementary material for: Not‐so‐simple nephrectomy: Comparative analysis of radical and simple nephrectomy in a high‐volume tertiary referral center
Source: Int J Urol. 2023 Nov 6;31(2):160–8. doi: 10.1111/iju.15330 (PMC11524099; doi:10.1111/iju.15330)
Supplement: Supplementary file 1 — Table S1–S5 [file IJU-31-160-s001.docx]

**Not So Simple Nephrectomy: Comparative Analysis of Radical & Simple Nephrectomy in a Tertiary Centre**

**Supplementary Material**

| Supplementary Table 1: Details of post-operative complications in the RN and SN groups by events (N=474) | | | | | | |
| --- | --- | --- | --- | --- | --- | --- |
|  | RN (n_1_=344) | n_2_ | (%) | SN (n_1_=129) | n_2_ | (%) |
| Clavien 1 | Wound infection (8), Retention (6), nausea/vomiting (5), ileus (4), hyponatremia (3) ,hematoma (2), surgical subcutaneous emphysema (2), incisional hernia (1),hematuria (1), blurred vision (1), abdominal pain (1), shoulder pain (1), pyrexia (1), upper extremity numbness (1), lower extremity numbness (1) | 38 | 53.5% | Wound pain (7), lower extremity numbness/pain (7), wound infection (4), ileus (4), nausea/vomiting (4), periumbilical pain/numbness (4), flank pain (3), upper extremity pain/numbness (2), chest pain (1), urinary pain (1), neuropraxia (1) incisional hernia (1), surgical subcutaneous emphysema (1), pyrexia (1) , wound dehiscence irrigation (1), erythematous rash (1), hematuria (1), hematoma (1), hyponatremia (1) | 46 | 70% |
| Clavien 2 | Wound infection antibiotics (6), blood transfusion (5), HAP (4), Ileus NG management (4), wound dehiscence antibiotics (2), delirium (2), hemorrhage drain (1), hernia surgical repair (1) | 25 | 35.2% | Wound infection antibiotics/ anti-fungal (5), Ileus NG management (2), HAP (2), wound dehiscence (2), pyrexia-antibiotics (2), acute gout of right hallux and metatarsals-colchine (1), wound discharge (1), acute confusion (1) | 16 | 22.2% |
| Clavien 3 | Hematoma requiring evacuation (2), Vasovagal syncope requiring pacemaker (2), Pneumothorax (1) | 5 | 7% | Sigmoidoscopy E.U.A. (1) | 1 | 1.5% |
| Clavien 4 | Metabolic/respiratory acidosis (1), sepsis (1), respiratory support ventilator (1) | 3 | 4.2% | Urosepsis (1), stroke (1) | 2 | 3% |
| Clavien 5 | - | 0 | 0% | Death-ischemic colitis (1) | 1 | 1.5% |
| Total |  | **71** |  |  | **66** |  |
| *Note: n_1_=number of cases, n_2_=number of events/ complications, RN=radical nephrectomy, SN=simple nephrectomy.* | | | | | | |

3

| Supplementary Table 2: Perioperative outcomes of SN and RN groups according to tumour size | | | | | | | | | | | | |
| --- | --- | --- | --- | --- | --- | --- | --- | --- | --- | --- | --- | --- |
|  | **SN (n=129)** | | **RN Group 1 (n=201)** | | **SN vs. RN1 P-value** | **RN Group 2 (n=94)** | | **SN vs. RN2**  **P-value** | **RN Group 3 (n=49)** | | **SN vs. RN3 P-value** | |
| Median operative time, minutes (Range) | 121 | (65-265) | 120 | (49-250) | **<0.001***** | 120 | (60-240) | 0.717 | 150 | (70-300) | 0.066 |  |
| Median length of stay, nights (Range) | 2 | (0-41) | 2 | (0-30) | **0.014*** | 2 | (1-21) | 0.294 | 3 | (1-27) | 0.136 |  |
| Median Blood Loss, ml (Range) | 40 | (0-1300) | 20 | (0-2000) | 0.186 | 50 | (0-2500) | 0.221 | 200 | (5-2000) | **<0.001***** | |
| Conversions, % (n) | 2% | (3) | 0% | (1) | 0.304 | 4% | (4) | 0.457 | 6% | (3) | 0.348 |  |
| HDU/ICU Admissions, % (n) | 6% | (7) | 5% | (10) | 0.800 | 10% | (9) | 0.294 | 8% | (4) | 0.731 |  |
| Readmissions, % (n) | 6% | (6) | 2% | (5) | 0.189 | 2% | (2) | 0.281 | 2% | (1) | 0.427 |  |
| Intraoperative, % (n) | 4% | (5) | 2% | (5) | 0.511 | 1% | (1) | 0.235 | 6% | (3) | 0.691 |  |
| Postoperative Complications, % (n) |  |  |  |  |  |  |  |  |  |  |  |  |
| Minor Complications  (Clavien grade 1-2) | 35% | (46) | 16% | (32) | **<0.001***** | 16% | (15) | **0.001**** | 27% | (13) | 0.261 |  |
| Major Complications  (Clavien grade 3-5) | 4% | (5) | 1% | (2) | 0.323 | 3% | (3) | 0.794 | 6% | (3) | 1.000 |  |
| Overall | 39% | (51) | 17% | (34) | **<0.001***** | 19% | (18) | **0.039*** | 33% | (16) | 0.532 |  |
| *P-value ≤0.05 was considered significant. Note: RN=radical nephrectomy, SN=simple nephrectomy. *=p<0.05; **=p<0.01; ***= p <0.001.* | | | | | | | | | | | |  |

| Supplementary Table 3: Perioperative outcomes of SN and RN by surgical approach | | | | | |
| --- | --- | --- | --- | --- | --- |
|  | **MI RN (n=313)** | | **MI SN (n=123)** | | **P-value** |
| Median operative time, minutes (Range) | 120 | (49-270) | 120 | (65-265) | **0.010** |
| Median length of stay, nights (Range) | 2 | (0-16) | 2 | (0-41) | **0.028** |
| Median Blood Loss, ml (Range) | 20 | (0-2,500) | 30 | (0-1,300) | 0.938 |
| Conversions, % (n) | 3% | (8) | 2% | (3) | 1.000 |
| HDU/ICU Admissions, % (n) | 4% | (12) | 3% | (4) | 1.000 |
| Readmissions, % (n) | 2% | (7) | 4% | (5) | 0.161 |
| Intraoperative, % (n) | 4% | (12) | 3% | (4) | 1.000 |
| Postoperative Complications, % (n) |  |  |  |  |  |
| Minor Complications  (Clavien grade 1-2) | 15% | (48) | 31% | (39) | **<0.001** |
| Major Complications  (Clavien grade 3-5) | 2% | (6) | 3% | (4) | 0.481 |
| Overall | 17% | (54) | 35% | (43) | **<0.001** |
| Transperitoneal approach, % (n) | 97% | (304) | 88% | (109) |  |
|  | **Open RN (n=34)** | | **Open SN (n=6)** | | **P-value** |
| Median operative time, minutes (Range) | 150 | (70-300) | 135 | (90-185) | 0.350 |
| Median length of stay, nights (Range) | 4 | (2-30) | 4 | (3-6) | 0.727 |
| Median Blood Loss, ml (Range) | 500 | (10-2,000) | 100 | (10-400) | **0.015** |
| Conversions, % (n) | - | - | - | - | - |
| HDU/ICU Admissions, % (n) | 35% | (12) | 0% | (0) | 0.153 |
| Readmissions, % (n) | 3% | (1) | 17% | (1) | 0.281 |
| Intraoperative, % (n) | 15% | (5) | 0% | (0) | 1.000 |
| Postoperative Complications, % (n) |  |  |  |  |  |
| Minor Complications  (Clavien grade 1-2) | 26% | (9) | 50% | (3) | 0.341 |
| Major Complications  (Clavien grade 3-5) | 6% | (2) | 0% | (0) | 1.000 |
| Overall | 32% | (11) | 50% | (3) | 0.740 |
| Transperitoneal approach,  % (n) | 97% | (33) | 67% | (4) |  |
| *Significance may be observed where median values are similar due to differences in data distribution between the groups. The Mann Whitney U test compares rank sums. P-value <0.05 was considered significant. Note: MI=minimally invasive, RN=radical nephrectomy, SN=simple nephrectomy. *=p<0.05; **=p<0.01; ***= p <0.001.* | | | | | |

| Supplementary Table 4: Patient and clinical characteristics in SN with Infective and Non-Infective Aetiology (N=130) | | | | | |
| --- | --- | --- | --- | --- | --- |
|  | **Infected SN**  **(n=50)** | | **Non-Infected SN (n=79)** | | **P-value** |
| Median age at surgery, years (Range) | 49 | (19-88) | 48 | (19-80) | 0.431 |
| Gender, % (n) |  |  |  |  |  |
| Male | 28% | (14) | 49% | (39) | **0.032*** |
| Female | 72% | (36) | 51% | (41) |  |
| ****Median ASA score (Range)**** | 2 | (1-3) | 2 | (1-3) | 0.765 |
| ****Laterality,**** % (n) |  |  |  |  |  |
| Left | 50% | (17) | 60% | (45) | 0.640 |
| Right | 50% | (17) | 40% | (30) |  |
| Median BMI (Range) | 25.8 | (15-41) | 27 | (16-58) | 0.990 |
| Median preoperative sCr, mg/dL (Range) | 86.5 | (55-623) | 89.5 | (53-856) | 0.168 |
| Median preoperative eGFR (Range) | 68 | (15-90) | 73 | (15-104) | 0.881 |
| Median preoperative INR (Range) | 1 | (0.9-1.1) | 1 | (0.9-1.3) | 0.804 |
| Initial MI approach, % (n) | 92% | (46) | 98% | (78) | 0.181 |
| Transperitoneal approach, % (n) | 88% | (44) | 86% | (69) | 0.773 |
| *P-value <0.05 was considered significant. Note: *=p<0.05; **=p<0.01; ***= p <0.001, RN=radical nephrectomy, SN=simple nephrectomy.* | | | | | |

| Supplementary Table 5: Perioperative outcomes of Infected and Non-Infected SN cases (N=130) | | | | | |
| --- | --- | --- | --- | --- | --- |
|  | **Infected SN (n=50)** | | **Non-Infected SN (n=79)** | | **P-value** |
| Median operative time, minutes (Range) | 150 | (82-265) | 120 | (65-260) | **<0.001***** |
| Median length of stay, nights (Range) | 3 | (0-41) | 2 | (1-16) | **0.005**** |
| Median Blood Loss, ml (Range) | 50 | (0-1,300) | 20 | (0-650) | **0.016*** |
| Conversions, % (n) | 2% | (1) | 3% | (2) | 1.000 |
| HDU/ICU Admissions % (n) | 12% | (6) | 1% | (1) | **0.019*** |
| Readmissions, % (n) | 10% | (5) | 2% | (1) | 0.204 |
| Intraoperative, % (n) | 2% | (1) | 6% | (4) | 0.403 |
| Postoperative Complications, % (n) |  |  |  |  |  |
| Minor Complications  (Clavien grade 1-2) | 36% | (18) | 35% | (28) | 0.908 |
| Major Complications  (Clavien grade 3-5) | 6% | (3) | 3% | (2) | 0.372 |
| Overall | 42% | (21) | 38% | (30) | 0.811 |
| Transperitoneal approach, % (n) | 88% | (44) | 86% | (69) | 0.773 |
| *P-value <0.05 was considered significant. Note: *=p<0.05; **=p<0.01; ***= p <0.001*, *RN=radical nephrectomy, SN=simple nephrectomy.* | | | | | |
